# Antioxidant, Analgesic, Anti-Inflammatory, and Hepatoprotective Effects of the Ethanol Extract of *Mahonia oiwakensis* Stem

**DOI:** 10.3390/ijms14022928

**Published:** 2013-01-30

**Authors:** Jung Chao, Jiunn-Wang Liao, Wen-Huang Peng, Meng-Shiou Lee, Li-Heng Pao, Hao-Yuan Cheng

**Affiliations:** 1 Department and Institute of Pharmacology, National Yang-Ming University, Taipei 112, Taiwan; E-Mail: rich720925@yahoo.com.tw; 2 Graduate Institute of Veterinary Pathobiology, National Chung Hsing University, Taichung 402, Taiwan; E-Mail: jwliao@dragon.nchu.edu.tw; 3 School of Chinese Pharmaceutical Sciences and Chinese Medicine Resources, College of Pharmacy, China Medical University, Taichung 404, Taiwan; E-Mails: whpeng@mail.cmu.edu.tw (W.-H.P.); leemengshiou@mail.cmu.edu.tw (M.-S.L.); 4 Department of biotechnology, Trans World University, Yunlin 640, Taiwan; E-Mail: paolh@ndmctsgh.edu.tw; 5 School of Pharmacy, National Defense Medical Center, Taipei 114, Taiwan; 6 Department of Nursing, Chung-Jen College of Nursing, Health Sciences and Management, Chiayi 600, Taiwan

**Keywords:** *Mahonia oiwakensis* Hayata, high-performance liquid chromatography, hepatoprotective effect, malondialdehyde

## Abstract

The aim of this study was to evaluate pharmacological properties of ethanol extracted from *Mahonia oiwakensis* Hayata stems (MOS_EtOH_). The pharmacological properties included antioxidant, analgesic, anti-inflammatory and hepatoprotective effects. The protoberberine alkaloid content of the MOS_EtOH_ was analyzed by high-performance liquid chromatography (HPLC). The results revealed that three alkaloids, berberine, palmatine and jatrorrhizine, could be identified. Moreover, the MOS_EtOH_ exhibited antioxidative activity using the DPPH assay (IC_50_, 0.743 mg/mL). The DPPH radical scavenging activity of MOS_EtOH_ was five times higher that that of vitamin C. MOS_EtOH_ was also found to inhibit pain induced by acetic acid, formalin, and carrageenan inflammation. Treatment with MOS_EtOH_ (100 and 500 mg/kg) or silymarin (200 mg/kg) decreased the serum alanine aminotransferase (ALT) and aspartate aminotransferase (AST) levels compared with the CCl_4_-treated group. Histological evaluation showed that MOS_EtOH_ reduced the degree of liver injury, including vacuolization, inflammation and necrosis of hepatocytes. The anti-inflammatory and hepatoprotective effect of MOS_EtOH_ were found to be related to the modulation of antioxidant enzyme activity in the liver and decreases in malondialdehyde (MDA) level and nitric oxide (NO) contents. Our findings suggest that MOS_EtOH_ has analgesic, anti-inflammatory and hepatoprotective effects. These effects support the use of MOS_EtOH_ for relieving pain and inflammation in folk medicine.

## 1. Introduction

Liver disorders are commonly caused by either toxic chemicals, drugs, or pathogen infection [1], and are considered extremely serious health problems in modern society. During chemical-induced liver injury, CCl_4_ metabolism begins with formation of the trichloromethyl free radical, CCl_3_, via the mixed function cytochrome P450 oxygenase system of the endoplasmic reticulum [2,3]. CCl_3_·can also react with oxygen to form the trichloromethylperoxyl radical, CCl_3_OO·, which is a highly reactive species [3]. Thus, CCl_3_OO·is more likely than CCl_3_·to abstract hydrogen from polyunsaturated fatty acids (PUFA), which leads to lipid peroxidation [4] and protein oxidation; these effects then cause hepatocellular membrane damage [5]. Additionally, CCl_4_-induced toxicity may stimulate endogenous reactive oxygen and nitrogen species production that seems to play an important role in the pathogenesis of hepatotoxicity. This process is followed by the release of inflammatory mediators from activated hepatic macrophages that are believed to promote CCl_4_-induced hepatic injury [2].

Many species of the *Mahonia* genus are considered to be medicinal plants [6–8]. Three species of *Mahonia* (Berberidaceae) grow in Taiwan [9]. The herbs *Mahonia japonica* and *Mahonia oiwakensis* are both native to Taiwan. All species of this genus in Taiwan are considered medicinal plants. *Mahonia oiwakensis* Hayata (MO), a popular folk medicine in Taiwan, is traditionally used by herbalists and Chinese doctors as a substitute for Phellodendri cortex, which is the bark of *Phellodendron amurense* or *Phellodendron chinese* (Rutaceae). The latter two preparations are known traditionally as antipyretic and analgesic drugs and also used for abdominal pain and diarrhea, inflammatory disorders (e.g., rheumarthritis), gastrointestinal disorders (e.g., dysentery and acute gastroenteritis), and liver disease (e.g., hepatitis) [9]. MO has been demonstrated to exhibit anti-tumor and anti-inflammatory activity [10,11]. Medical alcohol extracts of MO stems (MOS_EtOH_) are used to treat the common cold and enterogastritis in Taiwan [12,13]. However, scientific data on the chemical structure of the active ingredients and the hepatoprotective activity of MOS_EtOH_ are lacking.

This study was conducted to investigate the protoberberine alkaloid content of MOS_EtOH_ and its hepatoprotective activity. Protoberberine alkaloids are the dominant components found in *Berberis*, *Mahonia* and *Coptis* plant material [14]. Berberine and palmatine are the most medically significant protoberberine alkaloids. Therefore, the chemical components of MOS_EtOH_ was identified by high performance liquid chromatography (HPLC). Next, this study used *in vivo* and *in vitro* models to evaluate the antioxidative effect of MOS_EtOH_ and elucidate its possible hepatoprotective effects in rats. To examine the possible antioxidative activity of MOS_EtOH_, the 1,1-diphenyl-2-picrylhydrazyl (DPPH) radical scavenging assay was employed. Finally, the hepatoprotective effect of MOS_EtOH_ was determined using a CCl_4_-induced acute liver injury model. Silymarin, an effective therapic agent when there is CCl_4_-induced acute liver injury, was used as the therapeutic control.

## 2. Results and Discussion

### 2.1. Chromatographic Analysis of MOS_EtOH_

**Figure 1:**
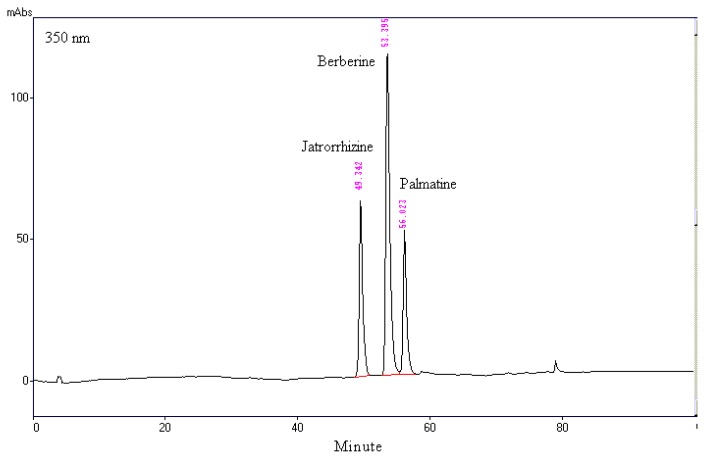
The HPLC chromatographic profile of MOS_EtOH_. λ = 350 nm showing the detection of jatrorrhizine, berberine and palmatine.

The major bioactive components in *Mahonia* plants are alkaloids [15]. The HPLC chromatogram showed that jatrorrhizine, berberine, and palmatine were the major components among organic molecules found in MOS_EtOH_, which had a maximum absorbance at 350 nm (Figure 1). This analytical result also indicated that the various different alkaloids present in MOS_EtOH_ were found at the following levels: berberine 135.84 ± 0.19 mg/g extract, palmatine 85.60 ± 0.03 mg/g extract, and jatrorrhizine 72.09 ± 0.46 mg/g extract.

### 2.2. The DPPH Radical Scavenging Activity of MOS_EtOH_

Evaluation of the antioxidative activity of MOS_EtOH_ was carried out using a DPPH radical-producing system. The IC_50_ of MOS_EtOH_ was 0.743 mg/mL. The IC_50_ of ascorbic acid (Vit. C) and 2,6-Di-*tert*-butyl-4-methylphenol (BHT) using the same system were 0.134 and 0.085 mg/mL (Table 1).

### 2.3. Toxicity Study

The acute toxicity of MOS_EtOH_ was evaluated using mice and doses up to 5000 mg/kg (p.o.) body weight administered for 72 h. MOS_EtOH_ did not cause any behavioral changes and no deaths occurred (data not shown). Thus the oral LD_50_ value of MOS_EtOH_ was greater than 5000 mg/kg body weight in mice and it can be considered to be practically a non-toxic substance. No toxicity symptoms were recorded. The lack of lethality means that the oral route of MOS_EtOH_ in mice cannot be determined to 5000 mg/kg.

### 2.4. Analgesic and Anti-Inflammatory Activity of MOS_EtOH_

**Figure 2:**
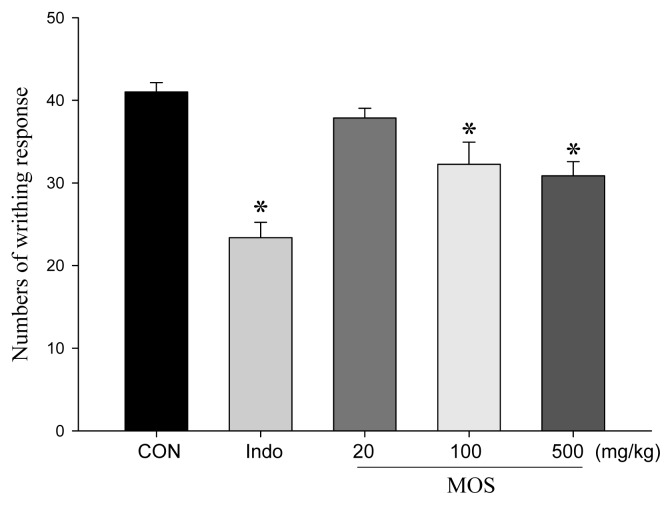
Analgesic effect of MOS_EtOH_ on acetic acid-induced writhing response in mice. Indomethacin (Indo 10 mg/kg) was use as a therapeutic control. The number of muscular contractions was evaluated as described in Section 2. Treatment of MOS_EtOH_ (20, 100 and 500 mg/kg) and Indo (10 mg/kg) showed that there was an inhibition of writhing number compared to the control. Each value represents as mean ± SEM (*n* = 10). *p < 0.05 as compared with the acetic acid-treated only group.

**Figure 3:**
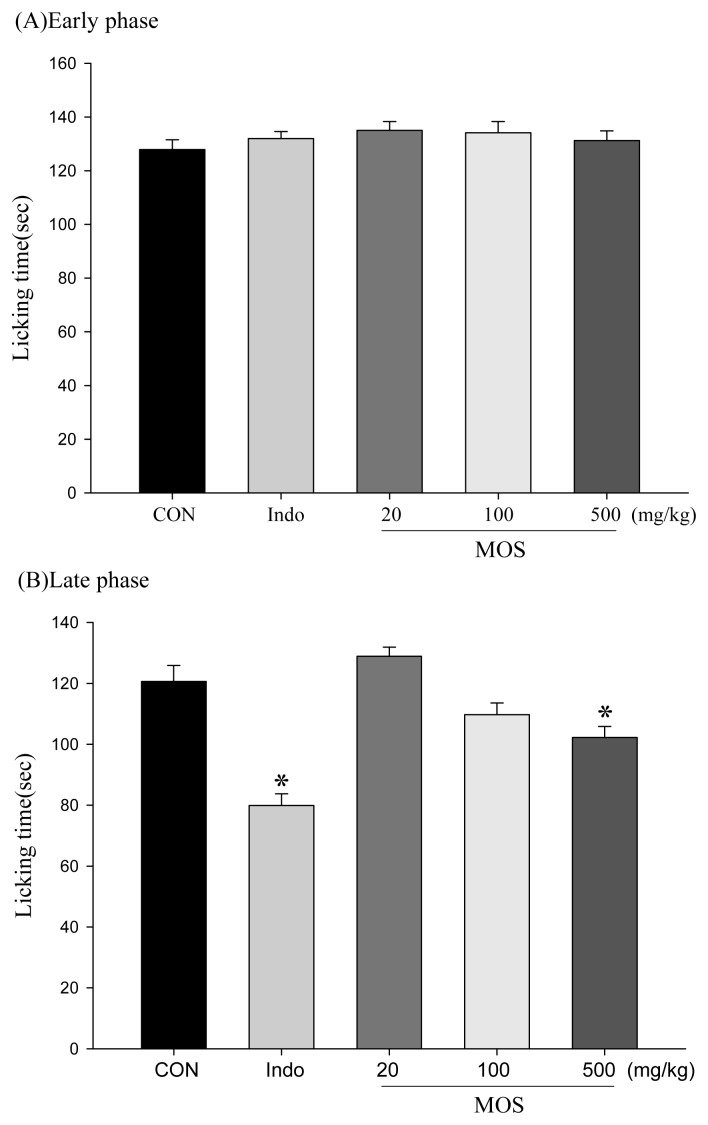
Effect of MOS_EtOH_ on (**A**) the early phase and (**B**) the late phase of the formalin test in mice. The index of pain (early phase and late phase) was evaluated as described in Section 2. MOS_EtOH_ (500 mg/kg) decreased the licking time during the late phase of formalin-induced pain test. Each value represents as mean ± SEM (*n* = 10). *p < 0.05 as compared with the formalin-treated only group.

MOS_EtOH_ was used to decrease the acetic acid-induced writhing responses in mice, which is an indication of the extract’s analgesic activity (Figure 2). Treatment with MOS_EtOH_ (100 and 500 mg/kg) or indomethacin (10 mg/kg) resulted in an inhibition of the writhing number compared to the control. Furthermore, there are no significant inhibitions during the early phase (Figure 3A). MOS_EtOH_ (500 mg/kg) decreased the licking time during the late phase of the formalin-induced pain test (Figure 3B, *p* < 0.05).

### 2.5. MOS_EtOH_-Inhibited Carrageenan-Induced Edema and Inflammation in Mice Paw Tissue

**Figure 4:**
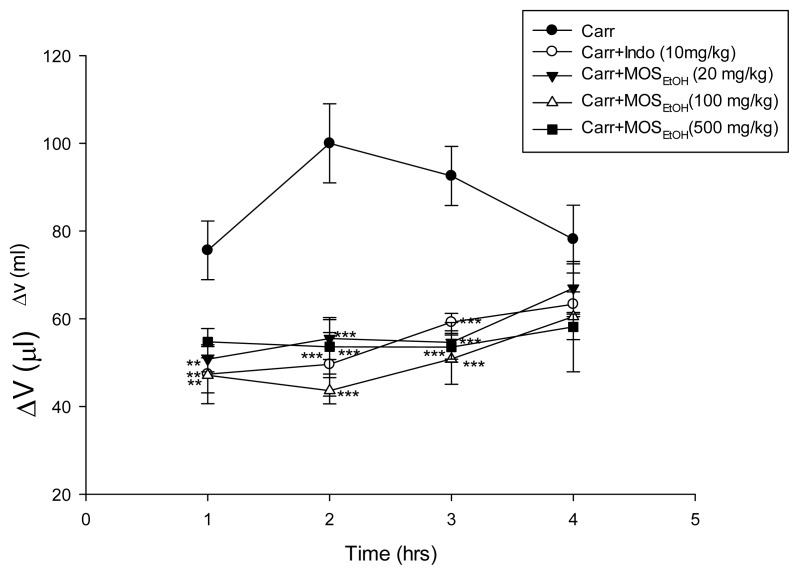
Inhibitory effects of MOS_EtOH_ on carrageenan-induced mice paw inflammation. Treatment with MOS_EtOH_ (20, 100 and 500 mg/kg) significantly reduced paw volume. Delta volume (ΔV) represents the degree of swelling of carrageenan-treated paw. Each value represents the mean ± SEM (*n* = 10). *p < 0.05 **p < 0.01, ***p < 0.001 as compared with the carrageenan-treated only group.

Carrageenan-induced mice paw edema is a biphasic process. During early hyperemia, which occurs 0–2 h after the carrageenan injection, there is a release of histamine, serotonin, and bradykinin that results in increased vascular permeability. The inflammatory edema reached its maximum level during the second hour and then begins to decline. In our study, paw edema was increased and reached a maximum at 2 h after carrageenan injection. Treatment with MOS_EtOH_ (20, 100 and 500 mg/kg) significantly reduced paw edema formation (*p* < 0.001) as shown in Figure 4. The inhibition rate at 2 h was 45% to 55% after treatment with MOS_EtOH_ (20, 100 and 500 mg/kg) or indomethacin.

### 2.6. Hepatoprotective Effect of MOS_EtOH_

**Figure 5:**
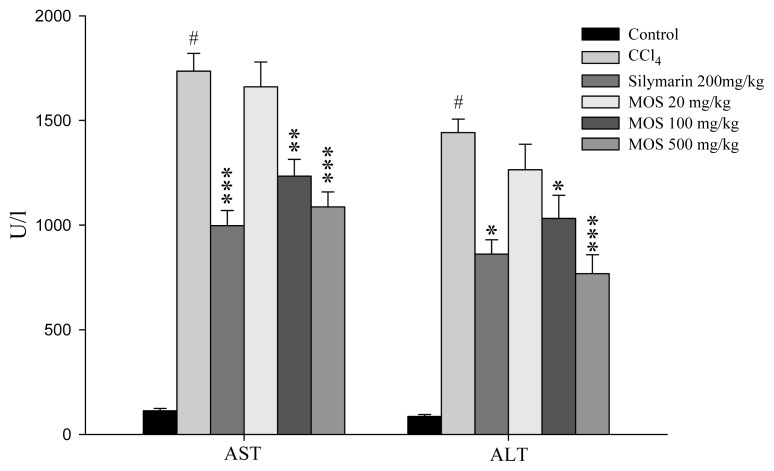
Effect of MOS_EtOH_ of serum AST and ALT in rats after intraperitoneally treated with CCl_4_. Values are mean ± SE (*n* = 6). ^#^ Significantly different from the control group (^###^*p* < 0.001); * Significantly different from the CCl_4_ group (*p < 0.05, **p < 0.01, ***p < 0.001).

Both AST and ALT are cytosolic enzymes present in hepatocytes and are released into circulation once cellular damage occurs. Figure 5 shows that the changes in serum AST and ALT activities for various group of animals. The serum levels of ALT and AST in untreated control group of animals were 112.5 ± 11.2 U/L and 85.7 ± 9.3 U/L, respectively. After CCl_4_ treatment, the serum ALT and AST activities increased by approximately 16.9 and 18.4-fold compared to the control group. In contrast, the serum AST and ALT levels showed a significantly lower increase when the rats were treated with MOS_EtOH_ compared to the CCl_4_ alone group of rats. Similar results were obtained when the rats were treated with silymarin, a known hepatoprotective chemical.

### 2.7. Histological Analysis

**Figure 6:**
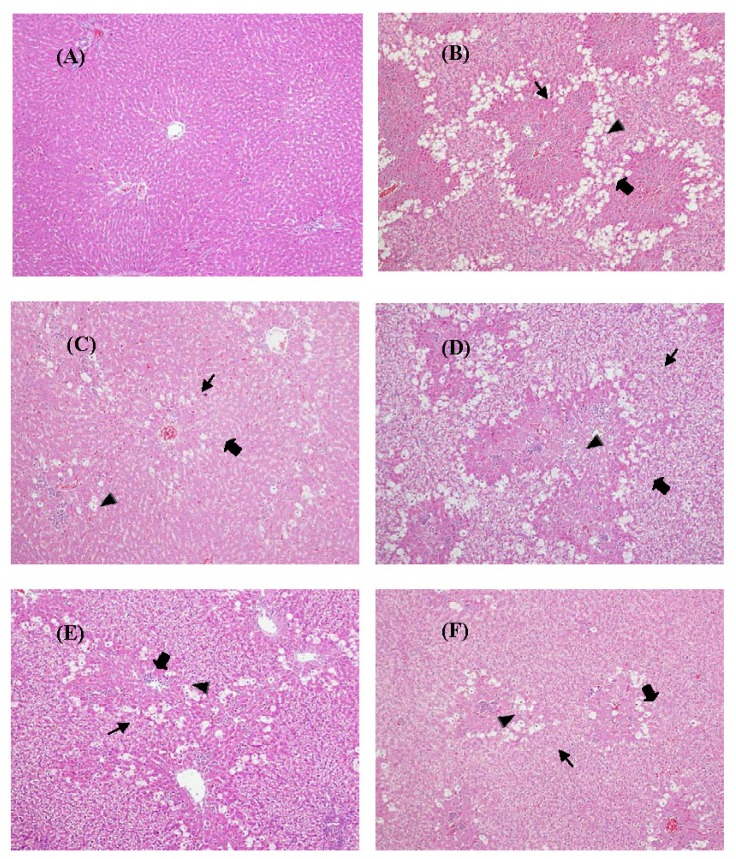
Effect of MOS_EtOH_ on CCl_4_-induced liver injury in rats. (**A**) The normal architecture of the liver of the control group; (**B**) The livers of animals treated with 50% CCl_4_ (1 mL kg/bw) showed marked cell necrosis (big arrow), vacuolization (small arrow) and inflammation with numerous neutrophilic infiltrations (small arrowhead). Fewer injured hepatocytes were found after treatment with sylimarin (200 mg/kg); (**C**) treatment with MOS_EtOH_ (20, 100 and 500 mg/kg; D, E, and F groups, respectively). Hematoxylin and eosin (H and E) stain, 100× magnification.

As compared with the control group of rats (Figure 6A), hepatic cell injury, including vacuolization, inflammation with neutrophilic infiltration and extended necrotic areas adjacent to portal triads, were present in the CCl_4_-treated group (Figure 6B). Treatment with silymarin significantly inhibited CCl_4_-induced hepatic injury (Figure 6C). The histopathological morphology of the MOS_EtOH_ treated groups (20, 100, and 500 mg/kg) are shown in Figure 6C–E. CCl_4_-induced acute liver damage in rats was attenuated after treatment with 100 and 500 mg/kg of MOS_EtOH_. Table 2 summarizes the data on the degree of liver damage induced by CCl_4_ for each group. The histoscores for vacuolization, inflammation and cellular necrosis of the livers were significantly higher after CCl_4_ treatment. In contrast, pretreatment with MOS_EtOH_ (500 mg/kg) and silymarin significantly reduced the injury histoscores of the livers from these groups.

### 2.8. Hepatic Lipid Peroxidation

Lipid peroxidation plays a critical role in CCl_4_-induced liver injury [17]. To evaluate the effect of MOS_EtOH_ pretreatment on CCl_4_-induced liver lipid peroxidation, malondialdehyde (MDA), the end product of lipid peroxidation, was monitored. It was found that administering CCl_4_ increased the hepatic level of MDA by about 1.3-fold compared to the control animals. This elevation was mitigated after administration of 100 or 500 mg/kg MOS_EtOH_ and after administration of silymarin (Table 3).

### 2.9. Antioxidative Enzyme Activity of Liver

Antioxidative enzyme activity in the liver was also analyzed. SOD activity in liver homogenates was decreased significantly after CCl_4_ administration (Table 3). SOD activity was increased significantly after pretreatment with either 100 mg/kg and 500 mg/kg of MOS_EtOH_ and after pretreatment with silymarin. Both GPx and GR activity in CCl_4_-treated group liver homogenates were significantly lower than those of the normal group (Table 3). Furthermore, pretreatment with MOS_EtOH_ increased the activities of both enzymes, as compared with the CCl_4_-treated group.

### 2.10. Hepatic NO Level Changes

The nitrite level in the liver was significantly elevated in the CCl_4_ administered group of rats compared to the control rats (Table 3). Only a slight increase in hepatic nitrite levels was found compared to the control group when there was pretreatment with either 100 mg/kg or 500 mg/kg MOS_EtOH_, and when there was pretreatment with silymarin; this indicates that the NO increase was attenuated by these pretreatments.

## 3. Discussion

Clinical medications are still unable to effectively control or treat liver disorders in humans. Patients suffering from liver diseases have started to try other methods of treatment, including complementary and alternative medicines (CAMs) to restore health; these treatments include herbal products [18,19]. Thus, numerous herbal medicines have been reported as being used for hepatoprotection. *Mahonia oiwakensis* Hayata, which is endemic plant in Taiwan, is a popular folk medicine that is traditionally used for treating inflammatory, gastrointestinal disorders and liver diseases in Taiwan. In this study, a single dose of 50% CCl_4_ at 1 mL/kg induced significant acute hepatic injury in rats, which was confirmed by the dramatic elevation in serum AST and ALT activity and the histological analysis. Additionally, CCl_4_ treatment also generated high MDA and NO levels and decreased SOD, GPx and GR levels; all of which are suggestive of oxidative stress. However, in this context, pretreatment with MOS_EtOH_ had a significant hepatoprotection with respect to CCl_4_-induced acute liver injury in rats.

Cell membrane permeability is associated with cell death and increased enzyme activity, both of which contribute to hepatic structural damage [2]. Both ALT and AST are enzymes that are correlated with injury to hepatocytes. Previous studies have demonstrated that CCl_4_ increases AST and ALT levels in serum [1]. In this study, the serum ALT and AST levels were increased markedly at 24 h after CCl_4_ injection; furthermore, these increases were attenuated by treatment with MOS_EtOH_ (100 and 500 mg/kg). These experimental results suggest that MOS_EtOH_ helps to maintain hepatocellular integrity and acts to prevent CCl_4_-induced hepatotoxicity; it does the above in a dose-dependent manner. The effect of MOS_EtOH_ was similar to silymarin, which has been shown previously to have a significant protective effect in rats. The hepatoprotective effects were confirmed by histological examinations. CCl_4_ causes a range of histological changes to the liver, including vacuole formation, inflammation, cellular swelling and extended necrotic areas from the central area to the portal triads. Previous reports have shown that rats treated with CCl_4_ undergo increased neutrophil infiltration into the liver cells as liver injury progresses. Furthermore, it has also been found that reactive oxygen species (ROS), such as superoxide radicals (O_2_·), are released from these activated neutrophils that have infiltrated into the liver of the CCl_4_-treated rats, which causes extensive liver cell necrosis [2,3]. Again, silymarin is known to exhibit a protective effect in terms of these changes. In the present study, these changes were also significantly attenuated by MOS_EtOH_ treatment.

Over-production of free radicals is toxic to hepatocytes and initiates ROS formation, which causes hepatocyte death and acute hepatic damage [20,21]. Based on this, antioxidative treatment has been proposed as a potential approach to preventing or attenuating toxic liver injury. Therefore, in this context, *in vivo* antioxidative activity was measured as part of this study by examining the activity levels of various antioxidative enzymes, namely SOD, GPx and GR. These antioxidative enzymes convert active oxygen molecules into non-toxic compounds and are easily inactivated by lipid peroxides or ROS during CCl_4_ exposure [2]. In this study, the experiment results showed that antioxidative enzyme activity levels in the MOS_EtOH_ and silymarin groups were increased significantly compared with those of the group treated with CCl_4_ only. Therefore, both MOS_EtOH_ and silymarin exert a protective effect against CCl_4_-induced hepatic injury and this is, at least in part, via their effect on hepatic antioxidative activity, which is able to reduce ROS production. The free radical, NO, is a highly reactive nitrogen species produced by parenchymal and nonparenchymal liver cells from l-arginine via nitric oxide synthase activity [22]. Thus, NO may contribute to the cytotoxic effect of neutrophils by forming peroxinitrite after it reacts with various ROS, particularly O_2_ [23,24]. In this study, only a slight increase in hepatic NO levels was noted in rats pretreated with MOS_EtOH_ or silymarin compared to rats administered with CCl_4_ alone, which showed a large increase. Suppression of NO production is likely due to the increases in SOD, GPx and GR activity induced by pretreatments.

Lipid peroxidation has been hypothesized to be a principal cause of CCl_4_-induced liver injury and can therefore be used as a marker of oxidative damage. The scavenging of free radicals is one of the major antioxidative mechanisms able to inhibit the chain reaction of lipid peroxidation. DPPH is known to abstract labile hydrogen [25]. The scavenging of DPPH radicals is thus related to the inhibition of lipid peroxidation [26]. The experiment results for DPPH scavenging activity suggests that MOS_EtOH_ is able to exert a free radical scavenging effect that could have a beneficial action against pathological alterations caused by free radicals generated by CCl_3_ and by lipid peroxidation. The final product of lipid peroxidation, MDA, is widely used as a marker of lipid peroxidation [27]. The fact that near-normal levels of hepatic MDA are maintained after MOS_EtOH_ pretreatment provides additional evidence indicating that MOS_EtOH_ has hepatoprotective activity. The results of the present study indicate that MOS_EtOH_ has antioxidative capacity, protects hepatocytes from oxidative stress, inhibits lipid peroxidation, reduces NO production, and enhances antioxidative activity in the rat livers that have been treated with CCl_4_.

Identification of the major compounds in a herb or a herbal preparation should prove helpful when elucidating pharmacological activity and the underlying mechanisms of action [8]. Protoberberine alkaloids are the predominant components of *Berberis*, *Mahonia* and *Coptis*, which have long histories as folk medicines [13]. The major compounds in MOS_EtOH_ were analyzed by HPLC. A number of major peaks were identified and these were found to be berberine, palmatine, and jatrorrhizine (Figure 1). Previous studies have demonstrated that berberine, palmatine, and jatrorrhizine possess significant anti-inflammatory and hepatoprotective activities [25,26]. Berberine, which is also present in *Coptidis* rhizoma and *Phellodendri* cortex, is known to scavenge O_2_·*in vitro* [28]. In this study, the analytical data indicates that the protoberberine alkaloids of berberine, palmatine, and jatrorrhizine make up 30% of the ingredients of MOS_EtOH_. Thus, the hepatoprotective activity of MOS_EtOH_ may relate to the presence of these alkaloids in MOS_EtOH_. Further in-depth studies are necessary in order to explore the mechanisms of action of MOS_EtOH_ and its individual components.

In nature, there are a great deal of natural plants with anti-inflammatory properties; flavonoids provide one of the more famous examples of a powerful ingredient found among these plants [29]. Flavonoids are polyphenolic compounds that are ubiquitous in nature and are categorized, according to chemical structure, into flavonols, flavones, flavanones, isoflavones, catechins, anthocyanidins and chalcones [30]. Over 4000 flavonoids have been identified, many of which occur in fruits (grape), vegetables (onions) and beverages (tea, wine and fruit drinks) [31,32]. Antioxidants are compounds that protect cells against the damaging effects of reactive oxygen species, such as singlet oxygen, superoxide, peroxyl radicals, hydroxyl radicals and peroxynitrite. An imbalance between antioxidants and reactive oxygen species results in oxidative stress, leading to cellular damage. Oxidative stress has been linked to cancer, aging, atherosclerosis, ischemic injury, inflammation and neurodegenerative diseases. The antioxidant activity of flavonoids depends on their molecular structure, and structural characteristics of certain flavonoids found in hops and beer confer surprisingly potent antioxidant activity exceeding that of red wine, tea, or soy.

Vegetables contain many physiologically active substances, and they can manipulate the growth of the plants and provide some pigments for the plants. These elements are called phytochemicals. Recently, epidemiological research indicated that fruits and vegetables in meals can reduce the risk of degenerative disease. This is the result of foods that might include plant phytochemicals. In the past, the related research of flavonoids was not valued. People usually get a small amount of flavonoids from the fruits and vegetables or in tea and alcoholic drinks [33], thus they have only paid attention to the ingredients in vegetables that offer some fructose and fibers, eschewing deeper investigation into the flavonoids’ metabolism, its decomposition, and its physiological effects after it has been metabolized. This area will be the focus of our future research.

## 4. Experimental Section

### 4.1. Chemicals

Berberine, palmatine, silymarin, Griess reagent and other chemicals were purchased from Sigma-Aldorich Chemical Co (St. Louis, MO, USA). Jatrorrhizine was purchased from the National Institute for the Control of Pharmaceutical and Biological Products (Beijing, China). CCl_4_ was purchased from Merck Co. (Merck KGaA, Darmstadt, Germany). The SOD, GPx and GR assay kits were purchased from Randox Laboratory Ltd. (London, UK). CCl_4_ was dissolved in olive oil as 50% (*v*/*v*) solution. Silymarin was suspended in 2% carboxymethyl cellulose. All other chemicals or reagents used were of analytical grade or HPLC grade.

### 4.2. Plant Source and Preparation of Plant Extract

The MO was collected from the Alishan mountainous area of Taiwan, and was identified by Chao-Lin Kuo, leader of the School of Chinese Medicine Resources (SCMR); a voucher specimen (Number: CMU MO 0722) was deposited at the SCMR. The crude MOS were sliced into small pieces, which were then dried in a circulating air stove and grounded up (453.4 g). Ten liters of ethanol were added to the dried powder. The MOS was extracted using 95% ethanol for 48 h four consecutive times. The filtrates were combined and concentrated under reduced pressure at 40 °C using a vacuum rotary evaporator in order to obtain MOS_EtOH_ extract. The yield ratio of the MOS_EtOH_ lyophilized extract (12.55 g) was 2.7%.

### 4.3. Chromatographic Identification of MOS_EtOH_

The HPLC system consisted of a Shimadzu (Kyoto, Japan) LC-10ATvp liquid chromatograph equipped with a DGU-14A degasser, an FCV-10ALvp low-pressure gradient flow control valve, a SIL-10ADvp auto injector, an SPD-M10Avp diode array detector, and an SCL-10Avp system controller. Peak areas were calculated using Shimadzu Class-LC10 software (Version 6.12 sp5). The column was a Phenomenex Synergi 4 Fusion-RP 80A column (250 × 4.6 mm). The gradient mobile phase was methanol (solvent A) and 1% triethylamine plus 1% acetic acid in water adjusted to pH 3.0 using phosphoric acid (solvent B). The sample was injected of 10 μL. The gradient profile was run at 1 mL min^−1^ over 100 min. The gradient program was as follow: 10–12 min 26% B isocratic, 12–14 min 26%–28% B, 14–19 min 28% B isocratic, 19–20 min 28%–34%, 20–38 min 34% B isocratic, 38–39 min 34%–42% B, 39–49 min 42% B isocratic, 49–50 min 42%–48% B, 50–59 min 48% B isocratic, 59–60 min 48%–55% B, 60–71 min 55%–70% B, 71–80 min 70% B isocratic, 80–100 min 70%–26% B. The solvent (mobile phase) was allowed to run for 3–5 min as the initial phase before injecting the next sample.

The peaks found in the MOS_EtOH_ samples were identified by comparison with the standard solutions of berberine, palmatine, and jatrorrhizine. The MOS_EtOH_ solutions were quantified by spiking with a known amount of standard and comparing the areas under the curve. The repeatability of the method was evaluated by injecting MOS_EtOH_ and the standard solutions three times, and the relative standard deviation (RSD) percentage was then calculated.

### 4.4. Determination of DPPH Radical Scavenging Ability

The effect of crude extracts and the positive controls (Vit. C, ascorbic acid; BHT, 2,6-Di-*tert*-butyl-4-methylphenol) on the DPPH radical scavenging ability was estimated according to a previously described method [34]. An aliquot (20 μL) of crude extracts at various concentrations was mixed with 100 mM Tris-HCl buffer (80 μL, pH 7.4) and then with 100 μL of the DPPH in ethanol to a final concentration of 250 μM. The mixture was shaken vigorously and left to stand at room temperature for 20 min in the dark. The absorbance at 517 nm of the reaction solution was measured spectrophotometrically. The percentage of DPPH decolorization of the samples was calculated according to the equation: % decolorization = [1 − (ABS sample/ABS control)] × 100. The half inhibitory concentration (IC_50_) value was the effective concentration at which DPPH radicals were scavenged by 50% and was obtained by interpolation from a linear regression analysis. A lower IC_50_ value indicates a greater antioxidative activity.

### 4.5. Animals

Male ICR mice (18~22 g) and Male Wistar rats (250~300 g) were obtained from the Animal Center of National Taiwan University (Taipei, Taiwan). They were housed in standard cages at a constant temperature of 22 °C ± 1 °C, relative humidity 55% ± 5% with 12 h light-dark cycle (08:00 to 20:00) for at least 1 week before experimentation began.

Animals used in this study were housed and cared for in accordance with the NIH Guide for the Care and Use of Laboratory Animals. The experimental protocol was approved by the Committee on Animal Research, China Medical University, under the code 2006-14-N. All tests were conducted under the guidelines of the International Association for the Study of Pain.

### 4.6. Acetic Acid-Induced Writhing Test

The writhing test in mice was conducted as described in the previous study [35]. Male ICR mice (ten per group) were fasted for 24 h before the experiment, but with free access to water. The writhes were induced by an intraperitoneal injection of 1.0% acetic acid in distilled water (0.1 mL/10 g body weight). Preliminary data showed that a dosage of 1.0 g/kg possesses maximum anti-inflammatory effects, and based on this, we chose three doses for subsequent animal experiments. Mice were administered orally with MOS_EtOH_ (20, 100 and 500 mg/kg) 60 min prior to chemical induction of writhes and the same volume of distilled water by oral administration as the vehicle control. Indomethacin (10 mg/kg, i.p) was administered 30 min prior to acetic acid injection. Mice were placed in an observation box separately and the number of writhing responses was counted over 10 min.

### 4.7. Formalin Test

The test was conducted according to the method described in the previous study [36]. Male ICR mice (ten per group) were fasted for 24 h before the experiment, but with free access to water. Twenty microliters of 5% formalin in distilled water was then injected subcutaneously into the right hind paw of mice to cause pain. Mice were administered orally with MOS_EtOH_ (20, 100 and 500 mg/kg) 60 min before formalin treatment and the same volume of distilled water by oral administration as the vehicle control. Indomethacin (10 mg/kg, i.p) was administered 30 min before formalin treatment. These mice were individually placed in a transparent Plexiglas cage (25 × 15 × 15 cm). The time spent licking and biting the injected paw was used as the index of pain and was recorded separately from 0 to 5 min as early phase or neurogenic pain and from 20 to 30 min as late phase or inflammatory pain [37].

### 4.8. Carrageenan-Induced Mice Paw Edema

This method was carried out previously described but with some modifications [38]. Male ICR mice (*N* = 10) were fasted for 24 h before the experiment with free access to water. The mice were injected subcutaneously with 50 μL of 1% carrageenan solution in normal saline (0.9% *w*/*v* NaCl) into the sub-plantar region of the right hind paw. Paw volume was measured using a plethysmometer immediately before injection and 1, 2, 3, and 4 h after the administration of the carrageenan. MOS_EtOH_ (20, 100 and 500 mg/kg) was administered at 120 min after carrageenan injection. Indomethacin (10 mg/kg, i.p), a therapeutic control, was administered at 150 min after carrageenan injection. The percent increase in paw volume was calculated and compared with the vehicle control.

### 4.9. Hepatoprotective Effect

For the dose selection of MOS_EtOH_, the acute oral toxicity of MOS_EtOH_ in rats was single gavaged at three levels of 500, 2500, and 5000 mg/kg body weight at a volume of 10 mL/kg. The results were observed for 48 h. Results revealed that MOS_EtOH_, up to 5000 mg/kg body weight, did not cause any significant behavioral changes and no mortality occurred. For the liver protection experiments, control and CCl_4_-treated rats were orally administered, and distilled water was used as the non-therapeutic control. The therapeutic control group weas given silymarin (200 mg/kg) orally for three consecutive days. The MOS_EtOH_ group of rats were orally administered MOS_EtOH_ (20, 100 and 500 mg/kg) for three consecutive days. One hour after the last administration of the experimental drugs, CCl_4_ (1 mL/kg, 50% *v*/*v*) was injected intraperitoneally into each group of rats, except for the control group. Control rats received a comparable volume of olive oil (i.p). Twenty-four hours after CCl_4_ injection, the rats were sacrificed under anesthesia and blood was collected for evaluation of the biochemical parameters (AST and ALT levels). Liver tissue was removed for histological evaluation, and parts of livers were also collected to allow the SOD, GPx and GRd, MDA and NO contents to be measured.

### 4.10. Histopathological Evaluation

All animals were subjected to necropsy at the end of experiment. The livers were observed grossly and then excised, blotted and weighed. The weights of liver are represented as percentage of final body weight. Tissues were fixed in 10% buffered formaldehyde solution and embedded in paraffin. The specimens were cut into 2 μm sections, stained with hematoxylin and eosin, and then examined by light microscopy.

### 4.11. Antioxidative Enzyme Activity Measurements

SOD enzyme activity was determined according to a previously described method at room temperature [36]. Tissue extract (100 μL) was added to 880 μL (0.05 M, pH 10.2, 0.1 mM EDTA) carbonate buffer. Epinephrine, 30 mM in 0.05% acetic acid (20 μL) was added to the mixture and the absorbance at 480 nm for 4 min was measured on a Hitachi U 2000 spectrophotometer. The enzyme activity is represented as the amount of enzyme that inhibits the oxidation of epinephrine by 50%, which is equal to 1 unit, and is expressed as U/mg protein.

The GPx enzyme activity was determined according to the method of Flohe and Gunzler [37] at 37 °C. A reaction mixture consisted of 500 μL phosphate buffer, 100 μL 0.01 M GR (reduced form), 100 μL 1.5 mM NADPH and 100 μL GR (0.24 units). Ttissue extract (100 μL) was added to the reaction mixture and incubated at 37 °C for 10 min. Then 50 μL of 12 mM *t*-butyl hydroperoxide was added to 450 μL of the tissue reaction mixture and the absorbance measured at 340 nm for 180 s. The molar extinction coefficient of 6.22 × 10^−3^ was used to determine the enzyme activity. One unit of activity is equal to the mM of NADPH oxidized min^−1^ per mg protein. NADPH (2 mM, 50 μL) in 10 mM Tris buffer (pH 7.0) was added to a cuvette containing 50 μL of GSSG (20 mM) in phosphate buffer. Tissue extract (100 μL) was added to the NADPH-GSSG buffered solution and the absorbance measured at 340 nm for 3 min. The molar extinction coefficient of 6.22 × 10^−3^ was used to determine GR enzyme activity. One unit of activity is equal to the mM of NADPH oxidized min^−1^ per mg protein and expressed as U/mg protein.

### 4.12. Hepatic MDA and NO Assay

The MDA levels in the liver tissue samples were evaluated by the thiobarbituric acid reacting substance (TRARS) method [39]. Briefly, MDA reacts with thiobarbituric acid at an acidic high temperature and forms a red-complex TBARS. The absorbance of TBARS was determined at 532 nm (Hitachi U 2000, Tokyo, Japan) and expressed as nmoL/mg protein.

Hepatic NO was measured according to the method of Moshage *et al.* [40]. For the nitrite determination, NO_3_^−^ was converted into nitrite by nitrate reductase enzymatic conversion; NO_2_^−^ was measured by the Griess reaction [41]. Values obtained by this procedure represent the sum of nitrite and nitrate (Hitachi U 2000, Tokyo, Japan) and expressed as μM/mg protein.

### 4.13. Statistical Analysis

All the data are shown as mean ± SE. The data in the present study were analyzed by one-way ANOVA followed by Bonferroni *post hoc* test. The exception was the liver histoscores, which were analyzed using nonparametric statistics. The criterion for statistical significance were ^#^*p* < 0.05, ^##^*p* < 0.01 and ^##^*p* < 0.001 for the comparison between CCl_4_ and the control groups, and * *p* < 0.05, ** *p* < 0.01 and *** *p* < 0.001 for the comparison between the MOS_EtOH_ and CCl_4_ groups.

## 5. Conclusions

This study demonstrates that MOS_EtOH_ exhibits antioxidant, analgesic, anti-inflammatory, and hepatoprotective effects. The anti-inflammatory and hepatoprotective effects of MOS_EtOH_ seem to be related to a modulation of antioxidant enzyme activity in the liver together with decreases in the malondialdehyde (MDA) level of the liver and nitric oxide (NO) content of the liver. The hepatoprotective mechanisms of MOS_EtOH_ when orally administered are via a preventive effect on liver injury progression in CCl_4_-treated rats; this seems to involve the maintaining of the liver antioxidative defense systems in addition to the scavenging of ROS and NO; these lead to an inhibition of lipid peroxidation. Thus it would seem that MOS_EtOH_ acts as a pharmacological agent that is able to prevent inflammatory and liver disorders.

## Figures and Tables

**Table 1:** The IC_50_ values of MOS_EtOH_ in DPPH radical scavenging activity.

Substance	DPPH radical scavenging activity (IC_50_, mg/mL)
MOS_EtOH_	0.743 ± 0.023
Vit. C	0.134 ± 0.015
BHT	0.085 ± 0.002

MOS_EtOH_: ethanol extracted from MO stems; DPPH: 1,1-diphenyl-2-picrylhydrazyl; Vit. C: ascorbic acid; BHT: 2,6-Di-*tert*-butyl-4-methylphenol. Values are mean ± SE (*n* = 3).

**Table 2:** Histoscores of livers treated with either silymarin or MOS_EtOH_ using the rat CCl_4_-induced hepatotoxicity assay.

Histo-grade	Group					

	Control	CCl_4_	Silymarin 200 mg/kg	MOS_EtOH_ (mg/kg)		

				20	100	500
Vacuolization	0	3.2 ± 0.2 ^##^	1.7 ± 0.2 ^**^	2.8 ± 0.3	2.5 ± 0.2	2.2 ± 0.2 ^**^
Inflammation	0	2.7 ± 0.2 ^##^	1.7 ± 0.2 *	2.3 ± 0.2	2.0 ± 0.3	1.5 ± 0.2 *
Cellular necrosis	0	3.5 ± 0.2 ^##^	1.5 ± 0.2 ^**^	3.0 ± 0.3	2.3 ± 0.2 ^**^	1.7 ± 0.2 ^**^

To quantify the histological indices for vacuolization and hepatocellular necrosis of liver, the slides were graded 0–4 according to the method of Knodell *et al.* [16]. The liver damage was graded 0–4 as following: 0 = no visible cell damage; 1 = slight (1%–25%); 2 = moderate (26%–50%); 3 = moderate/severe (51%–75%); 4 = severe/high (76%–100%). ^#^ Significantly different from the control group (^##^*p* < 0.01);

* Significantly different from the CCl_4_ group (^*^p < 0.05, ^**^p < 0.01); Values are mean ± SE (*n* = 6).

**Table 3:** Effect of MOS_EtOH_ on the level of serum NO, hepatic MDA and antioxidative enzymes in rats treated with CCl_4_.

Groups	MDA (nmoL/mg protein)	Activity (U/mg protein)			NO (μM/mg protein)

		SOD	GPx	GRd	
Control	1.30 ± 0.02	19.26 ± 0.61	9.05 ± 0.43	0.193 ± 0.006	3.12 ± 0.19
CCl_4_	2.11 ± 0.04 ^###^	14.19 ± 0.28 ^###^	4.31 ± 0.50 ^###^	0.145 ± 0.001 ^###^	7.71 ± 0.44 ^###^
Silymarin 200 mg/kg + CCl_4_	1.70 ± 0.04 ^***^	18.99 ± 0.43 ^***^	8.98 ± 0.65 ^***^	0.169 ± 0.002 ^***^	4.66 ± 0.33 ^***^
MOS_EtOH_ 20 mg/kg + CCl_4_	1.99 ± 0.06	15.03 ± 0.46	5.60 ± 0.51	0.158 ± 0.002	6.49 ± 0.33
MOS_EtOH_ 100 mg/kg + CCl_4_	1.88 ± 0.06 *	16.78 ± 0.42 ^**^	6.89 ± 0.16 ^**^	0.164 ± 0.003 ^**^	5.53 ± 0.40 ^**^
MOS_EtOH_ 500 mg/kg + CCl_4_	1.81 ± 0.05 ^**^	18.23 ± 0.43 ^***^	7.55 ± 0.15 ^***^	0.171 ± 0.003 ^***^	4.79 ± 0.32 ^***^

SOD: superoxide dismutase, GPx: glutathione peroxidase, GRd: glutathione reductase; MDA: malondialdehyde, NO: nitric oxide. ^#^ Significantly different from the control group. (^###^*p* < 0.001);

* Significantly different from the CCl_4_ group (^*^p < 0.05, ^**^p < 0.01, ^***^p < 0.001); Values are mean ± SE (*n* = 6).

## References

[1] Lee C.P.P., Shih H., Hsu C.L., Yen G.C. (2007). Hepatoprotection of tea seed oil (*Camellia oleifera* Abel.) against CCl_4_-induced oxidative damage in rats. Food Chem. Toxicol.

[2] Recknagel R.O., Glende E.A., Dolak J.A., Waller R.L. (1989). Mechanisms of carbon tetrachloride toxicity. Pharmacol. Ther.

[3] Weber L.W., Boll M., Stampfl A. (2003). Hepatotoxicity and mechanism of action of haloalkanes: Carbon tetrachloride as a toxicological model. Crit. Rev. Toxicol.

[4] Forni L.G., Packer J.E., Slater T.F., Willson R.L. (1983). Reaction of the trichloromethyl and halothane-derived peroxy radicals with unsaturated fatty acids: A pulse radiolysis study. Chem. Biol. Interact.

[5] McCay P.B., Lai E.K., Poyer J.L., DuBose C.M., Janzen E.G. (1984). Oxygen- and carbon-centered free radical formation during carbon tetrachloride metabolism. Observation of lipid radicals *in vivo* and *in vitro*. J. Biol. Chem.

[6] Gancevici G.G. (1990). Bioeffects of the plant Mahonia sempervirens. Arch. Roum. Pathol. Exp. Microbiol.

[7] Rohrer U., Kunz E.M., Lenkeit K., Schaffner W., Meyer J. (2007). Antimicrobial activity of *Mahonia aquifolium* and two of its alkaloids against oral bacteria. Schweiz. Monatsschr. Zahnmed.

[8] Tseng S.H., Chien T.Y., Tzeng C.F., Lin Y.H., Wu C.H., Wang C.C. (2007). Prevention of hepatic oxidative injury by Xiao-Chen-Chi-Tang in mice. J. Ethnopharmacol.

[9] Kan W.S. (1993). Pharmaceutical Botany.

[10] Chao J., Lu T.C., Liao J.W., Huang T.H., Lee M.S., Cheng H.Y., Ho L.K., Kuo C.L., Peng W.H. (2009). Analgesic and anti-inflammatory activities of ethanol root extract of *Mahonia oiwakensis* in mice. J. Ethnopharmacol.

[11] Wong B.S., Hsiao Y.C., Lin T.W., Chen K.S., Chen P.N., Kuo W.H., Chu S.C., Hsieh Y.S. (2009). The *in vitro* and *in vivo* apoptotic effects of *Mahonia oiwakensis* on human lung cancer cells. Chem. Biol. Interact.

[12] Kan W.S. (1980). Manual of Medicinal Plants in Taiwan (*I*).

[13] Luo Y.C., Yeh L.F., Zhang K.C., Lu F.Y. (2007). Ethnobotany in North Tsou tribe. Nat. Conserv. Q.

[14] Rackova L., Majekova M., Kost’alova D., Stefek M. (2004). Antiradical and antioxidant activities of alkaloids isolated from *Mahonia aquifolium* structural aspects. Bioorg. Med. Chem.

[15] Moshage H., Kok B., Huizenga J.R., Jansen P.L. (1995). Nitrite and nitrate determinations in plasma: A critical evaluation. Clin. Chem.

[16] Knodell R.G., Ishak K.G., Black W.C., Chen T.S., Craig R., Kaplowitz N., Kiernan T.W., Wollman J. (1981). Formulation and application of a numerical scoring system for assessing histological activity in asymptomatic chronic active hepatitis. Hepatology.

[17] Green L.C., Wagner D.A., Glogowski J., Skipper P.L., Wishnok J.S., Tannenbaum S.R. (1982). Analysis of nitrate, nitrite, and [15N] nitrate in biological fluids. Anal. Biochem.

[18] Basu S. (2003). Carbon tetrachloride-induced lipid peroxidation: Eicosanoid formation and their regulation by antioxidant nutrients. Toxicology.

[19] Seeff L.B., Lindsay K.L., Bacon B.R., Kresina T.F., Hoofnagle J.H. (2001). Complementary and alternative medicine in chronic liver disease. Hepatology.

[20] Strader D.B., Bacon B.R., Lindsay K.L., La Brecque D.R., Morgan T., Wright E.C., Allen J., Khokar M.F., Hoofnagle J.H., Seeff L.B. (2002). Use of complementary and alternative medicine in patients with liver disease. Am. J. Gastroenterol.

[21] Bissell D.M., Gores G.J., Laskina D.L., Hoofnagle J.H. (2001). Drug-induced liver injury: Mechanisms and test systems. Hepatology.

[22] Aeschke H., Gores G.J., Cederbaum A.I., Hinson J.A., Pessayre D., Lemasters J.J. (2002). Mechanisms of hepatotoxicity. Toxicol. Sci.

[23] Eller D.A., de Vera M.E., Russell D.A., Shapiro R.A., Nussler A.K., Simmons R.L., Billiar T.R. (1995). A central role for IL-1β in the *in vitro* and *in vivo* regulation of hepatic inducible nitric oxide synthase. IL-1β induces hepatic nitric oxide synthesis. J. Immunol.

[24] Rodenas J., Mitjavila M.T., Carbonell T. (1995). Simultaneous generation of nitric oxide and superoxide by inflammatory cells in rats. Free Radic. Biol. Med.

[25] Smith J.A. (1994). Neutrophils, host defense, and inflammation: A double-edged sword. J. Leukoc. Biol.

[26] Matsubara N., Fuchimoto S., Iwagaki H., Nonaka Y., Kimura T., Kashino H., Edamatsu R., Hiramatsu M., Orita K. (1991). The possible involvement of free radical scavenging properties in the actions of cytokines. Res. Commun. Chem. Pathol. Pharmacol.

[27] Ratty A.K., Sunamoto J., Das N.P. (1988). Interaction of flavonoids with 1,1-diphenyl-2-picrylhydrazyl free radical, liposomal membranes and soybean lipoxygenase-1. Biochem. Pharmacol.

[28] Ohkawa H., Ohishi N., Yagi K. (1979). Assay for lipid peroxides in animal tissues by thiobarbituric acid reaction. Anal. Biochem.

[29] Rodrigues A.S., Perez-Gregorio M.R., Garcia-Falcon M.S., Simal-Gandara J., Almeida D.P.F. (2011). Effect of meteorological conditions on antioxidant flavonoids in Portuguese cultivars of white and red onions. Food Chem.

[30] Rodrigues A.S., Perez-Gregorio M.R., Garcia-Falcon M.S., Simal-Gandara J. (2009). Effect of curing and cooking on flavonols and anthocyanins in traditional varieties of onion bulbs. Food Res. Int.

[31] Perez-Gregorio M.R., Regueiro J., Gonzalez-Barreiro C., Rial-Otero R., Simal-Gandara J. (2011). Changes in antioxidant flavonoids during freeze-drying of red onions and subsequent storage. Food Control.

[32] Perez-Lammela C., Garcia-Falcon M.S., Simal-Gandara J., Orriols-Fernandez I. (2007). Influence of grape variety, vine system and enological treatments on the colour stability of young red wines. Food Chem.

[33] Alonso Garcia A., Cancho Grande B., Simal-Gandara J. (2004). Development of a rapid method based on solid-phase extraction and liquid chromatography with ultraviolet detection for the determination of polyphenols in alcohol-free beers. J. Chromatogr. A.

[34] Yamaguchi T., Takamura H., Matoba T., Terao J. (1998). HPLC method for evaluation of the free radical-scavenging activity of foods by using 1,1-diphenyl-2-picrylhydrazyl. Biosci. Biotechnol. Biochem.

[35] Chiu Y.J., Huang T.H., Chiu S.H., Lu T.C., Chen T.W., Peng W.H., Chen C.Y. (2012). Analgesic and anti-inflammatory activities of the extract from *Plectranthus amboinicus* (Lour.) spreng. both *in vitro* and *in vivo* animal models. Evid. Based Complement. Alternat. Med.

[36] Mitchell J.R., Jollow D.J., Potter W.Z., Gillette J.R., Brodie B.B. (1973). Acetaminophen-induced hepatic necrosis. IV. Protective role of glutathione. J. Pharmacol. Exp. Ther.

[37] Hunskaar S.O., Berge G., Hole K. (1985). Antinociceptive effects of orphenadrine citrate in mice. J. Ethnopharmacol.

[38] Zimmermann M. (1983). Ethical guidelines for investigations of experimental pain in conscious animals. Pain.

[39] Lu T.C., Ko Y.Z., Huang H.W., Huang Y.C., Lin Y.C., Peng W.H. (2007). Analgesic and anti-inflammatory activities of aqueous extract from *Glycine tomentella* root in mice. J. Ethnopharmacol.

[40] Flohe L., Gunzler W.A. (1984). Assays of glutathione peroxidase. Methods Enzymol.

[41] Carlberg I., Mannervik B. (1985). Glutathione reductase. Methods Enzymol.

